# Microfilarial reduction following ProHeart® 6 and ProHeart® SR-12 treatment in dogs experimentally inoculated with a resistant isolate of *Dirofilaria immitis*

**DOI:** 10.1186/s13071-017-2430-z

**Published:** 2017-11-09

**Authors:** Tom L. McTier, Aleah Pullins, Gregory A. Inskeep, Genevieve Gagnon, Huihao Fan, Adam Schoell, Tara Bidgood, Joyce Login, Patrick Meeus

**Affiliations:** 1 Zoetis, Veterinary Medicine Research and Development, 333 Portage Street, Kalamazoo, MI 49007 USA; 2 Veterinary Operations, Zoetis, 10 Sylvan Way, Parsippany, NJ 07054 USA

**Keywords:** ProHeart® 6, ProHeart® SR-12, Moxidectin, Macrocyclic lactone, Canine heartworm, *Dirofilaria immitis*, Microfilaria, Resistance

## Abstract

**Background:**

Emerging resistance of heartworms (*Dirofilaria immitis*) to macrocyclic lactone (ML) preventives is an increasing concern for veterinarians, pet owners and animal health companies that supply heartworm preventives, with recent reports of resistant isolates identified from the Mississippi Delta region of the United States. Products that are effective in eliminating microfilariae (MF) in dogs harboring resistant heartworm infections could be important in reducing the spread of heartworm resistance. The current study was conducted to investigate the potential for ProHeart® 6 (PH 6; Zoetis) and ProHeart® SR-12 (PH 12; Zoetis) to reduce MF in dogs experimentally inoculated with an isolate of *D. immitis* (ZoeMo-2012) confirmed to be resistant to MLs.

**Methods:**

Twenty-three dogs with preexisting heartworm infections (via surgical transplantation) were randomly allocated to four groups based on pretreatment (Day −14) MF counts. On Day 0, dogs received a subcutaneous injection of either saline (placebo-treated control, 6 dogs), PH 6 (0.17 mg/kg, 6 dogs), PH 12 (0.5 mg/kg, 5 dogs) or a single oral dose of moxidectin powder in a gelatin capsule (0.25 mg/kg, 6 dogs). All dogs were bled for MF counts (modified Knott’s test) on Days 0 (pretreatment), 1, 3, 7, 14, 21, 28, 42, 56, and 84. Dogs in control and PH 6 groups were also bled for MF counts on Days 112, 140, and 168. No adverse events associated with treatment were observed for any dog.

**Results:**

Average reductions in MF counts compared with controls for PH 6 were 9.7% on Day 1, increasing to 75.0% on Day 7, and further to 86.5% on Day 28. On Day 42, average MF reduction increased to 90.3%. Reductions increased further over the next several months with reductions of 91.3, 96.8, 96.6, and 98.9% on Days 56, 84, 112, and 140, respectively. On Day 168, the reduction was 99.3% (*P* < 0.0001). Average reductions in MF counts compared with controls for PH 12 were 20.9% on Day 1, increasing to 78.9% on Day 7, and further to 91.2% on Day 28. On Day 84, the reduction was 96.9%. For dogs receiving a single oral moxidectin (0.25 mg/kg) on Day 0, reductions in MF were 86.3% on Day 1 and fluctuated between 74.4 and 83.6% through Day 28. On Days 42 and 56, percentage reductions were 87.1 and 81.8%, respectively, and 92.6% at the final time point (Day 84).

**Conclusion:**

Both PH 6 and PH 12 were highly effective in reducing the MF levels of a confirmed ML-resistant heartworm isolate following a single dose.

## Background

Resistance of the canine heartworm, *Dirofilaria immitis,* to macrocyclic lactone (ML) preventive medications is now well documented [1–3]. Much is yet to be learned, however, about the nature and extent of this resistance in natural populations. A better understanding of the epidemiology and transmission dynamics of heartworm in areas where resistant strains of heartworm occur is necessary to estimate how this resistance may spread. Work is being undertaken to gain more information on these factors, but the progress is somewhat slow due to the complicated and multifaceted aspects of this inquiry [4, 5]. Baseline survey data on the current level and geographical distribution of resistance are required, along with factors underlying and contributing to the development and the spread of this resistance.

Reducing the availability of resistant microfilariae in nature for transmission to other competent hosts would be beneficial in reducing the spread of resistant heartworms. Recent publications have shown that several ML-based products that were originally potent microfilaricides failed to substantially reduce or clear some dogs of circulating microfilariae (MF) later identified as resistant [3, 6, 7]. However, there have been no reports on the microfilaricidal activity of ProHeart® 6 (PH 6) (Zoetis) or ProHeart® SR-12 (PH 12) (Zoetis) against resistant heartworm MF. The purpose of the current investigation was to determine the activity of PH 6 and PH 12 in reducing microfilarial levels in dogs surgically implanted with a resistant isolate (ZoeMO-2012) of *D. immitis (*see “*D. immitis* Isolate” following).

## Methods

### Ethical approval

The study was a masked, negative placebo-controlled, randomized laboratory efficacy study conducted in Michigan, USA. Study procedures were conducted in accordance with the VICH guidelines (GL19) [8]. Masking of the study was assured through the separation of functions. All personnel conducting observations or animal care or performing infestations and counts were masked to treatment allocation. The protocol for this study was approved by the Zoetis Institutional Animal Care and Use Committee (IACUC), and the study was conducted in accordance with state and national/international regulations regarding animal welfare.

### Animals

Twenty-five (25) dogs with preexisting adult heartworm infections, established via surgical transplantation (10 pairs of adult heartworms) [9] with an isolate (ZoeMO-2012 isolate), confirmed to be resistant to MLs [10, 11] were available for this study. The dogs had not been treated with a monthly oral preventive dose of a ML for at least 180 days before inoculation with heartworms. Dogs were identified individually by unique numeric ear tattoo or digital microchip. For at least 14 days prior to treatment, dogs were acclimated to the study facilities. For the duration of the study, dogs were housed in individual enclosures, which prevented physical contact with adjacent animals. Dogs were offered water ad libitum and were fed an appropriate standard commercial canine diet. Prior to inclusion in the study, dogs were examined for overall general physical health and study suitability.

### *D. immitis* isolate

The heartworm isolate used in this study was designated ZoeMo-2012. A blood sample was collected from a heartworm-positive dog, originally from Pittsfield, Illinois, USA, on December 4, 2012, which was used to infect *Aedes aegypti* (Liverpool strain) mosquitoes. On December 19, 2012, two dogs were each inoculated with 50 infective larvae (L3) that developed in these mosquitoes. On July 18, 2013, these two dogs were positive for MF on a modified Knott’s test, validating passage of this isolate. This isolate was taken from the same parent dog from which the original JYD-34 isolate had been taken 2.5 years earlier (John McCall, personal oral communication, August 2013). The dog had been maintained in mosquito-proof quarters with no additional macrocyclic lactones administered during the intervening time from JYD-34 isolation to ZoeMo-2012 isolation.

### Design

On Day −14, 2 weeks prior to treatment, dogs were tested for adult *D. immitis* antigen and were examined by the modified Knott’s method for MF. Animals with MF counts >1000 MF/mL and that had positive results on a heartworm antigen (DiroCHEK®; Zoetis) test on Day −14 were included in the study. Two of the 25 dogs available for the study did not meet the minimum requirement for MF on Day −14 and were excluded from the study. The remaining 23 animals were randomly allocated to four treatments based on MF counts as follows: placebo-treated control (six dogs), ProHeart® 6 (PH 6, six dogs), ProHeart® SR-12 (PH 12, five dogs), and oral moxidectin (six dogs/group).

### Treatment

On Day −7, each dog was weighed and given a physical examination. On Day 0, control dogs were administered a single subcutaneous (SC) saline injection (0.05 mL/kg); PH 6 dogs were administered a single SC injection of 0.17 mg/kg body weight (BW) according to label directions; PH 12 dogs were administered a single SC dose of 0.5 mg/kg BW according to label directions; and dogs in the oral moxidectin group were given a single oral dose of 0.25 mg/kg BW of moxidectin in a gelatin capsule (hydroxypropyl methylcellulose moxidectin powder). Without any previous data available on which to select an oral dose of moxidectin for microfilaricidal efficacy against a resistant isolate, the authors chose the highest dose that was estimated could safely be given to MF-positive dogs.

#### Microfilariae and adult heartworm counts

Dogs in control and PH 6 groups were bled for MF counts on Days 0 (pretreatment), 1, 3, 7, 14, 21, 28, 42, 56, 84, 112, 140, and 168. Dogs treated with PH 12 and oral moxidectin at 0.25 mg/kg were bled for MF counts on Days 0, 1, 3, 7, 14, 21, 28, 42, 56, and 84.

Three control dogs and the dogs in the PH 12 and oral moxidectin groups were necropsied on Day 84 for adult heartworm counts, and the three remaining control dogs and the dogs in the PH 6 group were necropsied for adult worm counts on Day 168. Due to study management contraints and the priority of collecting PH 6 data, data collection for PH 12 was concluded on Day 84. The three control dogs selected to be necropsied on day 84 were randomly selected prior to Day 84.

For recovery of adult heartworms, at the time of euthanasia each dog was given approximately 1 mL of heparin (1000 USP units/mL) intravenously, prior to a lethal- dose euthanasia solution. After euthanasia, the pleural and peritoneal cavities were examined for adult *D. immitis* worms, and the posterior and anterior venae cavae were clamped before removal of the heart and lungs. The precava, right atrium, right ventricle and pulmonary arteries (including those coursing through the lungs) were dissected and examined for worms. The number and viability of worms recovered from each dog were determined.

#### Animal observations

Dogs were observed regularly for general health and for adverse events associated with treatment. General health observations (GHOs) were conducted once daily during the acclimation period, twice daily on Days 0–3 (>12 h apart) and for the remainder of the study (>5 h apart). GHOs were conducted when clinical observations (COs) were not performed and included but were not limited to: observations of general physical appearance and behavior, abnormalities of food and water consumption, vomiting/regurgitation and appearance of urine and feces. A suitably experienced veterinarian conducted COs according to the following schedule: Day −14 (± 2 days); Day 0 immediately prior to treatment and 2 to 4 h posttreatment; once daily on Days 1 to 7; once weekly during Days 8 to 84; and once per month during Days 85 to 168. COs included but were not limited to: overall condition, general attitude and cognition, evaluation for vomiting, abnormal feces, abnormal urine, abnormal appetite and any type of hypersensitivity reactions (anaphylaxis, shock, collapse, respiratory distress, depression or fever).

## Results

No adverse events associated with treatment with either PH 6, PH 12, or oral moxidectin (0.25 mg/kg) were observed for any dog. Mean MF counts in control dogs were 15,000.0 MF/mL on Day 0, with a somewhat variable but general overall trend toward increasing levels of MF as the study progressed (Table 1). On Day 7, control MF counts dropped to a mean of 12,566.7 MF/mL (the lowest of the study) before rebounding to 14,756.7 MF/mL on Day 14 and further to 23,133.3 MF/mL on Day 112 and finally to 26,633.3 MF/mL on Day 168. It should be noted that all six control dogs were used for percentage reduction calculation for the first 84 days, and for the final 80 days (to Day 168) the remaining three matched control dogs were used for efficacy calculation.

**Table 1:** Mean microfilariae (MF) counts (per mL) after treatment with a single SC dose of either PH 6 (0.17 mg/kg) or PH 12 (0.5 mg/kg) or a single oral treatment with moxidectin (0.25 mg/kg) on Day 0

Day of Study													
Test Group	0	1	3	7	14	21	28	42	56	84	112	140	168
Control	15,000.0	18,308.3	16,488.3	12,566.7	14,756.7	19,400.0	20,383.3	15,830.0	16,381.7	20,816.7	23,133.3	18,633.3	26,633.3
PH 6	13,916.7	16,531.7	11,796.7	3141.7	4358.3	4318.3	2755.0	1530.0^*^	1421.7	675.0^*^	780.0^*^	203.7^*^	176.8^*^
PH 12	11,520.0	14,480.0	6330.0	2656.0	2486.0	2918.0	1786.0	916.2^*^	997.0^*^	646.0^*^	NA	NA	NA
Oral Moxidectin (0.25 mg/kg)	12,033.3	2501.7	3138.3	3213.3	2830.0	3833.3	3333.3	2040.0	2983.3	1531.7	NA	NA	NA

^*^Significantly different from control mean (*P* < 0.05)

**Fig. 1:**
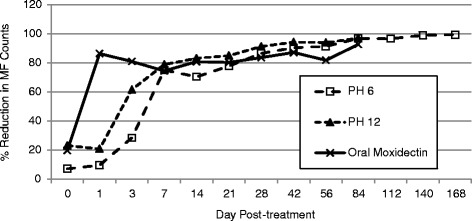
Percentage reduction in microfilarial counts of a resistant isolate of *Dirofilaria immitis* (ZoeMo-2012) after treatment with a single SC dose of either PH 6 (0.17 mg/kg) or PH 12 (0.5 mg/kg) or a single oral treatment with moxidectin (0.25 mg/kg) compared with controls

Microfilarial counts for both PH 6 and PH 12 decreased gradually over time compared with those in control animals (Table 1). Average reductions in MF counts for PH 6 were 9.7% on Day 1, increasing to 28.5 and 75.0% on Day 3 and 7, respectively, and further to 86.5% on Day 28 (Table 2; Fig. 1). On Day 42, average MF reduction increased to >90.3%. Reductions increased further over the next several months with reductions of 91.3, 96.8, 96.6, and 98.9% on Days 56, 84, 112, and 140, respectively. At the final time point (Day 168), the reduction was 99.3%. Mean MF counts decreased from 13,916.7 (range: 800–34,500 MF/mL) on Day 0 to 176.8 on Day 168 (range: 1–490 MF/mL) in PH 6 dogs (Table 1). None of the PH 6 dogs was ever negative for MF, but one dog had a single MF on Day 168. Mean MF counts for PH 6 were significantly lower (*P* < 0.05) than control counts on Days 42, 84, 112, 140, and 168 (Table 2).

**Table 2:** Percentage reduction in mean microfilaria (MF) counts (compared with control) after treatment with a single SC dose of either PH 6 (0.17 mg/kg) or PH 12 (0.5 mg/kg) or a single oral treatment with moxidectin (0.25 mg/kg) on Day 0

Day of Study													
Test Group	0	1	3	7	14	21	28	42	56	84	112	140	168
PH 6	7.2	9.7	28.5	75.0	70.5	77.7	86.5	90.3	91.3	96.8	96.6	98.9	99.3
PH 12	23.2	20.9	61.6	78.9	83.2	85.0	91.2	94.2	93.9	96.9	ND	ND	ND
Oral Moxidectin (0.25 mg/kg)	19.8	86.3	81.0	74.4	80.8	80.2	83.6	87.1	81.8	92.6	ND	ND	ND

Reductions in MF counts were similar for PH 6 and PH 12 (Table 2; Fig. 1). Average reductions in MF counts compared with controls for PH 12 were 20.9% on Day 1, increasing to 61.6 and 78.9% on Days 3 and 7, respectively and further to 91.2% on Day 28. On Days 42 and 56, MF reductions were 94.2 and 93.9%, respectively, increasing slightly to 96.9% on Day 84 (the last time point for this group). For PH 12, MF counts were similar to those for PH 6, with initial mean counts at 11,520 MF/mL and decreasing to 646 MF/mL (675 MF/mL for PH 6) on Day 84. One dog had 0 MF on Days 56 and 84, despite having a high initial MF level (31,000 MF/mL on Day 0). Mean MF counts for PH 12 were significantly lower (*P* < 0.05) than control counts on Days 42, 56, and 84 (Table 2).

For dogs receiving a single oral moxidectin treatment (0.25 mg/kg) on Day 0, MF levels decreased more rapidly immediately after treatment compared to those for PH 6 and PH 12, with a mean reduction of 86.3% on Day 1. However furthur reductions were not observed during the following 2 months. On Day 84 (the final time point), the reduction was 92.6%. In addition, mean MF counts for oral moxidectin were not significantly lower (*P* < 0.05) than control counts on any of the count days.

The dogs treated with PH 12 and oral moxidectin and three control dogs were necropsied on Day 84 and the dogs treated with PH 6 and three controls were necropsied on Day Day 168 for recovery of adult heartworms (Table 3). A mean of 15.3 and 16.7 of the 20 worms/dog initially transplanted were recovered from the control and PH 6-treated, respectively, indicating no effect of PH 6 on adult heartworms over the 168-day study period. The numbers of heartworms in the PH 12 and oral moxidectin groups were reduced by 19 and 14%, respectively; and there did appear to be a greater reduction of the female worms (30 and 23%, respectively) compared with controls. However, these reductions were not statistically different (*P* > 0.05).

**Table 3:** Mean adult heartworm counts and percentage reductions at necropsy (compared with control) after treatment with a single SC dose of either PH 6 (0.17 mg/kg) or PH 12 (0.5 mg/kg) or a single oral treatment with moxidectin (0.25 mg/kg) on Day 0

Test Group	Values	Live Adult D. immitis Counts^3^		
		Males	Females	Total
Control^1^	Mean Mean % Reduction	7.7	7.7	15.3^a^
PH 6^1^		8.2	8.5	16.7^a^
		0%	0%	0%
Control^2^	Mean Mean % Reduction	8.7	10.0	18.7^a^
PH 12^2^		7.7	6.5	14.9^a^
		5%	30%	19%
Control^2^	Mean Mean % Reduction	8.7	10.0	18.7^a^
Oral Moxidectin (0.25 mg/kg) ^2^		8.3	7.7	16.0^a^
		4%	23%	14%

^1^Necropsied on Day 168

^2^Necropsied on Day 84

^3^Surgically transplanted with 10 pairs of heartworms ~12 weeks prior to treatment

^a^Means with the same superscripts and not statistically different (*P* > 0.05)

## Discussion

These are the first reported data showing the microfilaricidal effects of ProHeart® (PH 6 and PH 12) on a resistant isolate of *D. immitis* in the dog. Lack of efficacy of approved monthly preventive products containing selamectin, milbemycin oxime and ivermectin against the JYD-34 isolate has been previously demonstrated [2], suggesting that this isolate is resistant to approved doses of some MLs. Additional genetic analysis of markers associated with resistance [1] along with evidence of heritable resistance characteristics [3] have allowed us to confirm this resistance.

Subsequently, we have confirmed that both the JYD-34 and ZoeMo-2012 isolates, as well as several other recent field isolates (ZoeLA and AMAL), are ML-resistant through both phenotypic testing in dogs using an oral preventive dose (3 μg/kg) of moxidectin [10] and by genetic testing of the isolates using genetic marker analysis [11].

All MLs have demonstrated microfilaricidal effects on susceptible microfilariae [12–23], and one of these MLs in a product (Advantage Multi®; Bayer) with the active ingredient moxidectin (2.5%, topical) has a claim for this indication [24]. However, none of these products, except ProHeart® 6 and ProHeart® SR-12 reported here, has demonstrated microfilaricidal potency against a resistant heartworm isolate. A product that has the ability to reduce MF levels of resistant strains substantially could be useful in reducing the overall risk of transmission of these strains in a natural population where resistant heartworm strains occur.

Additional surveillance work on the baseline prevalence of heartworm resistance, along with periodic monitoring across the United States, is needed to understand more completely the risk that populations of animals have to exposure to resistant heartworms; and some of this work has already begun [4].

## Conclusions

Both PH 6 and PH 12 reduced microfilarial levels of a confirmed resistant isolate of *D. immitis* (ZoeMO-12) by >92% at 84 days and for PH 6 by >99% at 168 days after a single subcutaneous injection.

## Abbreviations

COs: Clinical observations
GHOs: General health observations
MF: Microfilariae
ML: Macrocyclic lactone
SC: Subcutaneous
